# Label-Free Multiple Reaction Monitoring, a Promising Method for Quantification Analyses of Specific Proteins in Bacteria

**DOI:** 10.3390/ijms21144924

**Published:** 2020-07-12

**Authors:** Anna A. Toymentseva, Anastasia O. Koryagina, Alexander V. Laikov, Margarita R. Sharipova

**Affiliations:** Department of Microbiology, Kazan (Volga Region) Federal University, 18 Kremlyovskaya Street, 420008 Kazan, Russia; tojmencevaaa@mail.ru (A.A.T.); alexander.laikov@yandex.ru (A.V.L.); marsharipova@gmail.com (M.R.S.)

**Keywords:** mass spectrometry, multiple reaction monitoring (MRM), protein quantification, subtilisin-like protease (AprBp), glutamyl endopeptidase (GseBp), *Bacillus pumilus*

## Abstract

*Bacillus subtilis* produces eight industrially important exo-proteases. For the detection of proteases, the activity- and antibody-based assays are normally used. Current activity-based assays require expensive multiplex chemical substrates which allow specificity determination of each enzyme. In this study, we provide evidences pertaining to the usefulness of the label-free multiple reaction monitoring (MRM) assay for a rapid identification and quantitation of specific proteins in bacteria. We used wild-type *B. pumilus* cells producing at least two serine proteases, subtilisin-like protease (AprBp) and glutamyl endopeptidase (GseBp), as well as optimized recombinant *B. subtilis* cells containing the same protease genes under control of the LIKE expression system. The Skyline software was used for the selection of three specific proteotypic peptides and their fragment ions for quantification and confirmation of AprBp and GseBp in complex mixtures. MRM indicated that the production of AprBp and GseBp exo-enzymes were respectively 0.9- and 26.6-fold higher in the culture medium of *B. pumilus* strain in comparison to the recombinant *B. subtilis* strains carrying optimized LIKE expression systems under identical conditions. The developed procedure in this study is fast, easy to perform and dependable. Additionally, it achieves accurate proteins identification and quantification in complex mixture.

## 1. Introduction

The broad-spectrum physiological function of proteases includes maturation of proteins and their ultimate destruction [1]. Due to their panoramic applications, proteases have become the most important industrial enzymes involved in nearly 60% of the total worldwide enzyme sales [2,3]. Hence, the production of proteases remains an important task in biotechnology. The Gram-positive non-pathogenic bacterium *Bacillus subtilis* falls under the category of hosts, generally recognized as safe (GRAS). Besides the absence of endotoxin (lipopolysaccharide, LPS) production, *B. subtilis* secrete eight different proteins, all of which belong to the groups of serine- and metalloproteases. Thus, different expression systems have been developed for *B. subtilis*, which facilitate well-controlled protein production and secretion [4,5]. These systems ensure strict gene regulation using inducible promoters, such as xylose-inducible or isopropyl β-galactopyranoside (IPTG)-inducible or autoinducible promoters; a typical example being the SigB-dependent promoter [6,7,8,9,10,11]. The LIKE expression system is one of the most effective expression systems that operates via the PliaI promoter [12]. PliaI is induced by a wide range of compounds including antibiotics (bacitracin, vancomycin and nisin), as well as different stress conditions, such as alkaline shock and stationary phase of the bacterial life cycle.

Accurate protein quantification is essential during the assessment of expression system efficiency. The yield of secreted proteins is usually measured and verified by methods, such as direct spectrophotometry, enzyme activity, western blotting analysis, real-time quantitative reverse transcription PCR (real-time qRT-PCR) and ELISA (enzyme-linked immunosorbent assay). Despite their simplicity, some of these methods lack accuracy and reliability, owing to indirect estimation of protein level. In most cases quantitative estimation of proteins is carried out using spectrophotometric means such as the Bradford protein assay, which is only feasible after purification. On the other hand, the reliable methods based on antibodies or substrates for individual enzymes tend to be laborious and/or expensive. The prospect of mass spectrometry in overcoming the drawbacks of other protein quantification methods is inarguable. Mass spectrometry provides accurate estimation of low-abundant compounds in complicated mixture (such as crude cell extract) and shows high analytical reproducibility. Multiple reaction monitoring (MRM)-based targeted quantitative assay is generally performed on a triple-quadrupole (QQQ) mass spectrometer. It requires the selection of a predefined precursor ion in the first quadrupole (Q1), which is then fragmented in the second quadrupole (Q2) that serves as a collision cell. Following fragmentation, a predefined set of fragment ions are filtered in the third quadrupole (Q3), which are then transmitted to a detector. The peak area of each of the precursor transitions ion is integrated and used for quantification [13]. Precursor ions (proteotypic peptides) for analyzed proteins and their fragment ions must be reproducibly generated during sample preparation. To achieve high accuracy in protein quantitation a stable isotope labeled peptides can be used as internal standards in the MRM method. Isotopic labels can also be used to multiplex proteomic analysis, such as iTRAQ (isobaric tags for relative and absolute quantitation) for labeling peptides or SILAC for labeling proteins with amino acids in cell culture. Such labeling significantly increases the analysis costs which relatively conserves the popularity of the label-free quantitative approach among proteomics methods [14,15,16].

The present study aimed at using label-free MRM assay for quantitative detection of two *B. pumilus* serine proteases. These enzymes exhibit different substrate specificity and were produced in varying amounts: 70% subtilisin-like protease (AprBp) and 10% glutamyl endopeptidase (GseBp) of total protein in culture medium [17,18]. Quantification of these proteases was carried out using activity determination. Since this approach ignores other proteases with similar substrate specificity, we conducted a comparative concentration evaluation of proteases of the original *B. pumilus* strain and *B. subtilis* recombinant strains carrying the same expression system. We were able to show that the label-free MRM approach can provide a rapid, robust and efficient verification/quantification of any protein in bacterial expression systems.

## 2. Results

### 2.1. Selection of Specific Peptides for Detection of AprBp and GseBp Proteins

To detect the AprBp and GseBp serine proteases expressed in *B. pumilus* and by the LIKE expression system in *B. subtilis*, specific proteotypic peptides and their fragment ions must primarily be selected. To date, advanced proteotypic peptide databases exist for human proteins (PeptideAtlas (http://www.peptideatlas.org/), GPM Proteomics Database (http://gpmdb.thegpm.org/), the National Institute of Standards and Technology (NIST, http://peptide.nist.gov/), PRIDE (https://www.ebi.ac.uk/pride/archive/), etc.). In contrast, bacterial proteotypic peptides databases have not well been developed and appear to contain information limited to certain bacteria (e.g., SRM-Atlas for *Mycobacterium tuberculosis*). The optimal choice of peptides and their transitions is crucial for the sensitivity and selectivity of MRM experiment. Typically, proteotypic peptides (i) consist of 7 to 25 residues, (ii) have double or triple charged *m/z* values within the mass range of the instrument, (iii) efficiently produced during enzymatic digestion and (iv) lack modification sites or amino acids prone to variable changing during the sample processing [19]. First, proteotypic peptides for AprBp and GseBp and their transitions were predicted by the Skyline software ver. 20.1 according to the above-mentioned selection criteria [20]. For this purpose, AprBp and GseBp proteins were digested (trypsinized) in silico and peptides (precursors) with their transitions were selected. The uncleaved sequences of AprBp and GseBp have 381 and 303 residues with a theoretical molecular weight of 27 and 23 kDa, respectively [21,22]. Amino acids 1–29 (for AprBp) and 1–26 (for GseBp) comprise signal peptides which are normally removed from the mature proteins by cleavage prior to secretion into cultivation media. Amino acids 30–107 (for AprBp) and 27–89 (for GseBp) comprise pro-peptides cleaved during protein processing resulting in the functional AprBp and GseBp proteins from amino acid 108–381 and 89–303, respectively. Peptides and transitions obtained with Skyline software are listed in Table S1. The recommended parameters were generated, and the MRM method was carried out on a QTRAP instrument.

**Figure 1:**
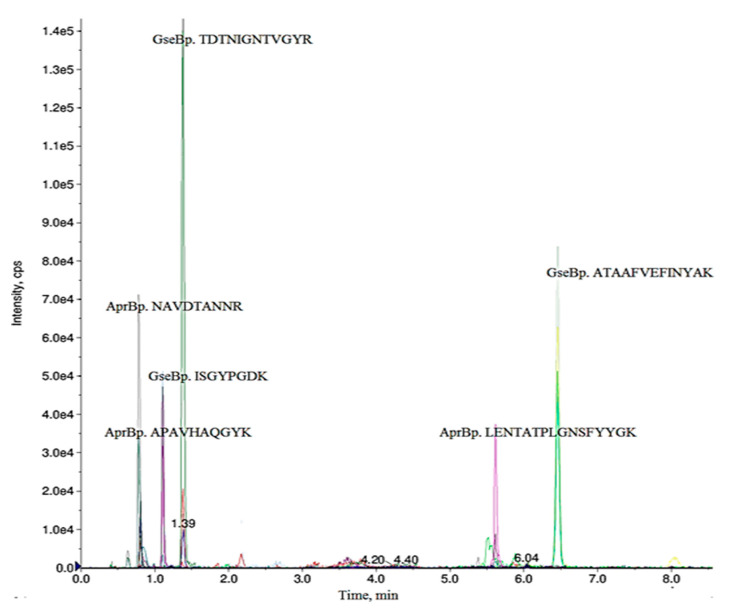
Ion chromatograms of selected MRM transitions monitored for serine proteases AprBp and GseBp. Three different peptides were considered for each protein confirmation. The MRM transitions 655.82/993.51 for GseBp peptide TDTNIGNTVGYR, 487.83/860.42 for AprBp peptide NAVDTANNR, with the best sensitivity and a lower interference/matrix effect were taken for further quantification in blind samples.

Ensuring the detectability, specificity and reproducibility of selected peptides for MRM experiment and their transitions in every sample is imperative. The validation of selected peptides and their transitions was performed using two extracellular (secretory) protein fractions, proteins from the *B. pumilus* 3–19 (positive control) and the *B. subtilis* AT1 (negative control). Extracellular protein fractions were collected, precipitated, run in the SDS-PAGE and trypsinized in the gel (Figure S1). Peptides were extracted from the gel and analyzed using LC-MRM-MS (by AB SCIEX QTRAP 6500 instrument) with a dwell time of 20 ms. For both proteases, three proteotypic peptides (each with best 2–4 transitions) showing higher intensity, stable retention time, easy fragmentation, high signal intensity of the fragment ions and symmetrical chromatographic peak shape were experimentally confirmed (Figure 1 and Table 1). For AprBp the precursor ion (Q1) NAVDTANNR (242–251 residues) with the most intense transition *m*/*z* 487.73 → 860.42 was selected for quantification. To quantify GseBp, the transition *m*/*z* 655.82 → 993.51 of the peptide TDTNIGNTVGYR (190–202 residues) was used.

### 2.2. Calibration Plots for Quantification of Serine Proteases AprBp and GseBp

**Figure 2:**
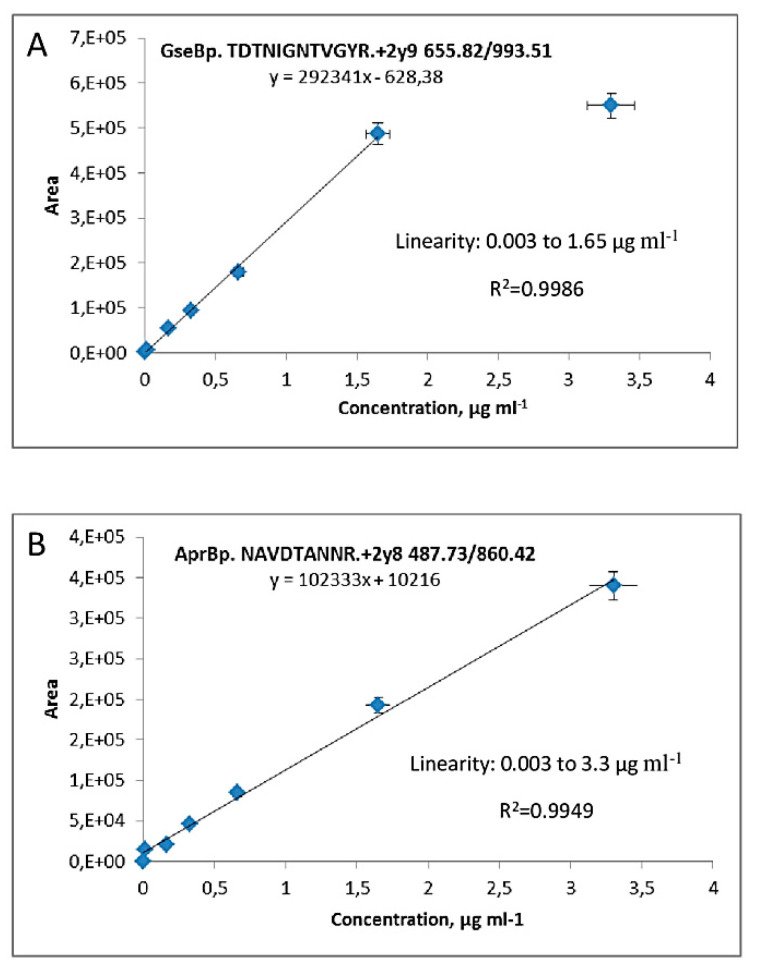
Calibration curve for serine proteases GseBp and AprBp by MRM transitions. The calibration plot for the selected transitions for (**A**) GseBp *m*/*z* 655.82 to *m*/*z* 993.51 of TDTNIGNTVGYR; (**B**) AprBp transition *m/z* 487.73 to *m/z* 860.42 NAVDTANNR. Exhibited linearity up to 1.65 µg mL^−1^ for GseBp, 3.3 µg mL^−1^ for AprBp.

A calibration curve using a highly intense transition for each target protein is vital for accurate protein quantification by MRM analysis. For this purpose, purified serine proteases with a degrees of protein purity of 757.11 for AprBp and 1257 for GseBp, were used to construct the calibration curve, basing on a range that us suitable for the protein concentration in the samples [21,22]. Purified enzymes (AprBp and GseBp) were added to the gel and separated via a one-dimensional SDS-PAGE. protein bands corresponding to proteases were cut from the gel and enzymatic digestion was performed. Extracted peptides were dissolved in a concentration of 3.3 μg mL^−1^ protein, which was taken as the maximal point on the calibration curve. A serial dilution from 0.0033 µg mL^−1^ to 3.3 µg mL^−1^ was prepared. The calibration plot for the TDTNIGNTVGYR+2y9 transition exhibited linearity of up to 1.65 µg mL^−1^ for GseBp protease with a lower limit of quantification at 0.003 µg mL^−1^ (Figure 2A). The NAVDTANNR+2y8 transition of AprBp exhibited linearity of up to 3.3 µg mL^−1^ with a lower limit of quantification at 0.0033 µg mL^−1^ (Figure 2B).

### 2.3. Quantification of AprBp and GseBp Serine Proteases by the Selected Peptides

To express the AprBp and GseBp genes for comparative MRM analysis, recombinant *B. subtilis* strains (MRB044, MRB046, MRB047, MRB049) were cultivated under two conditions; with or without the addition of the inducer bacitracin (50 µg mL^−1^) [12,23]. In addition, wild-type strain *B. pumilus* 3-19 was used as a positive control, while strain *B. subtilis* AT1 was used as a negative control. Strain *B. subtilis* AT1 containing pLIKE-rep plasmid was derived from a prototrophic *B. subtilis* BG2036 strain with knocked-out alkaline (AprE) and neutral (NprE) protease genes. All *B. subtilis* recombinant strains were derivatives of strain BG2036 as well. The extracellular fraction of secreted proteins was collected by TCA precipitation. One hundred micrograms of total precipitated proteins were separated by one-dimensional SDS-PAGE. The bands corresponding to the weight of the proteases were cut from gels and subjected to tryptic digestion. Finally, mass spectrometric analysis of extracted peptides was performed.

NAVDTANNR+2y8 of AprBp and TDTNIGNTVGYR+2y9 peptides of GseBp were not detected in culture medium of *B. subtilis* strain AT1 as expected (Table 2). In the culture medium of *B. pumilus* strain 3–19, three proteotypic peptides for each protease were detected. Concentrations of both proteins in the 50 mL supernatant of the *B. pumilus* 3–19 were 1.3 μg mL^−1^ and 1.63 μg mL^−1^ for GseBp and AprBp, respectively (Table 2).

The recombinant strain MRB044, in which AprBp protein was produced with a signal peptide SP_AprBp_, showed the most effective production of AprBp. In MRB044, there was a 60-fold increase in protein concentration after induction, while the addition of bacitracin led to a 6-fold increment in AprBp production in the MRB046 strain, expressing SP_Yngk_-AprBp. Nevertheless, the AprBp protein yield in MRB046 was 50-times less than strain MRB044. These results indicate that the substitution of the natural AprBp signal peptide with SP_Yngk_ does not enhance the AprBp secretion. On the contrary, the commutation of the natural GseBp signal peptide improved its yield. Inducing the LIKE expression system in *B. subtilis* strain MRB049, containing a GseBp protein with a recombinant SP_Yngk_, led to a 12-times enhanced production of GseBp in comparison with MRB047 (Table 2).

## 3. Discussion

MRM assays are usually used for the detection of special biomarkers, diagnosis [24,25,26,27] and disease status confirmation. They are likewise used for the detection of different chemical compounds in nature [28,29,30,31,32,33]. The application of MRM technology in proteomics is quite restricted due to high cost of labeled peptides for accurate quantitation. For this reason, label - free quantification does not reduce popularity implementation of this methodology has become possible also considering the availability of software tools for automatic peptide spectra prediction, as well as the optimization of chromatography-mass spectrometry and instrument tuning, in order to achieve the highest sensitivity, reproducibility and throughput for hundreds or thousands of samples. The high-throughput MRM method has a wide linear dynamic range of up to five orders of magnitude, very high precision, and is sufficiently sensitive in detecting ng/mL amounts of analytes in biologic fluids and cell or tissue protein extracts [34,35,36].

In the present study, we successfully implemented the targeted proteomics approach for quantitative detection of specific proteins (bacillary serine proteases), using non-labeled proteotypic peptides with their native sequences. To achieve high-level of protein secretion into culture medium the LIKE-expression system was optimized via heterologous SP. The choice of SP can significantly influence the secretion level of proteins and prevent the formation of inactive inclusion bodies; (B) *megaterium* is a well-known bacterium with high yield of exogenous secretion capacity. Stammen et al. (2010) demonstrated that the glycoside hydrolase SP of *B. megaterium* (Yngk) produces the highest secretion efficiency for the heterologous hydrolase of *Thermobifida fusca* (6-fold higher than its own signal peptide) [37]. Using the same approach, we conducted a comparative assessment of the overexpression of protease genes in a native *B. pumilus* and recombinant *B. subtilis* strains containing inducible LIKE expression systems. Serine proteases were *in silico* digested and fragmented to generate candidate transitions in the Skyline software. Skyline was also used to generate a method file with predicted values of collision energy and declustering potential. Thus, methods for quantifying proteases were rapidly generated using the Skyline software, which facilitated the simultaneous detection of both serine proteases. Subsequently, proteases from extracellular crude extract were loaded on the gel, protein bands were isolated trypsinized and followed by a UHPLC-triple quadrupole mass spectroscopic method, operated in MRM mode. We identified the calibration curve linearity for selected transitions (peptides–fragments ions). The low detection limit of sustained identification of AprBp and GseBp was three nanograms per milliliter. The effectiveness of the analysis varies for different proteins within the same sample. For instance, two nanograms per milliliter for apolipoprotein E and ng/mL for fibronectin were registered on similar LC-MS equipment [32]. Thus, the sensitivity and quality of MRM analysis depend on the complexity of samples (that determines matrix effects), the choice of most appropriate peptide and transition. Transitions chosen in our work were stable and reproducible throughout the experiment. As a result, our method demonstrates a high level of performance together with reduced cost. Although the protein amount recovered via the in-gel digestion method is lower than its original amount (by up to 70%–80%) [38], the label-free method is suitable for comparing relative protein levels in different conditions or growth phase. To better understand the ability of the bacterial strain in proteases production, it is important to know the amount and the activity of studied proteins. Application of the conventional proteases activity methods has a major disadvantage in that, other background proteases may have an influence the final results. For this reason, relative proteases quantification remains helpful in comprehending the amount of protein and directs researchers towards optimization of other methods of protein production assessment and quantitation. Here, we demonstrated that AprBp and GseBp are present at approximately equal concentrations in the supernatant of the native strain *B. pumilus* 3–19. Previously, amounts of these enzymes were always estimated based on their activities [17,18] (Figure S2). Considering the activity indices, we anticipated higher amounts of AprBp and lower amounts of GseBp proteases. The current data demonstrates that there is no direct correlation between protein amount and protease activity. Moreover, we observed that promoter and signal peptide substitution for AprBp protease and the expression host inversely affects the protein amount (the concentration of protein was decreased). However, replacing the promoter and signal peptide and host for GseBp tends to increase protein concentrations. These results coupled with previous data on protease activity obtained on the base of the LIKE expression system [12] evidently demonstrate that the expression system does not guarantee strong protein expression, secretion and enhanced activity level. For every heterologous target, it is crucial to choose individual expression systems and secretion conditions. Such results may have several explanations: (A) inclusion bodies formation, (B) differences in Sec system translocation machinery and maturation process, as well as varying native folding mechanisms of *B. pumilus* and *B. subtilis*. Each of these steps is imperative (bottle neck) and can have an effect on proper protein formation. Although the method described in this study is cost-effective and does not require tag-coupling, it is sensitive and suitable enough for the detection of small amounts of target proteins in complex protein mixtures. We suppose that the developed method based on the label-free MRM has prospective applications in measuring the expression of gene-encoded proteins, such as membrane proteins, which are purified with difficulty.

## 4. Materials and Methods

### 4.1. Materials

Sequencing grade modified trypsin was obtained from Promega (#V5111), whereas dithiothreitol (DTT), iodoacetamide (IAA), trichloroacetic acid (TCA), trifluoroacetic acid (TFA), formic acid (FA) and ammonium bicarbonate for trypsin digestion were purchased from Sigma-Aldrich (Dorset, UK). All solvents (acetonitrile, water and formic acid) were LC–MS grade. Erythromycin, lincomycin, streptomycin and bacitracin were likewise procured from Sigma-Aldrich (Dorset, UK).

### 4.2. Bacterial Strains and Growth Conditions

Bacterial strains were grown in lysogeny broth (LB) or on LB agar plates (per liter: 10 g tryptone, 5 g yeast extract, 5 g NaCl and 20 g agar for LB agar). Based on necessity, the media were supplemented with antibiotics: erythromycin (10 μg mL^−1^), lincomycin (25 μg mL^−1^) or bacitracin (50 μg mL^−1^, an inducer for P*_liaI_* promoter). All broth-cultured strains were incubated at 37 °C and 200 rpm aeration. The optical density (OD) of cell growth was determined by measuring at λ = 600 nm by xMark spectrophotometer (BioRad, Hercules, CA, USA). Streptomycin-resistant *B. pumilus* 3–19 strain producing the two proteases (AprBp, GseBp) and protease-deficient *B. subtilis* BG2036 strain were adopted as positive and negative controls, respectively. All bacterial strains used in this study are listed in Table 3.

### 4.3. Sample Preparation

For expression experiments, single colonies of *B. subtilis* hosts containing the recombinant constructions and *B. pumilus* 3–19 strain were inoculated into 5 mL LB and cultured overnight. Overnight cultures were diluted 1:100 in 50 mL of fresh LB and incubated in 500-mL flasks until an OD_600_ of 0.4 was obtained. A total of 50 µg mL^−1^ of bacitracin was added as an inducer to the medium of *B. subtilis* hosts carrying the recombinant genetic constructions, after which all bacterial cultures were incubated with vigorous shaking (200 rpm) for 24 h at 37 °C. To obtain extracellular (secretory) protein fraction, cells were harvested by centrifugation for 20 min at 5.000 rcf and the supernatant was filtered through a polyethersulfone syringe-driven filter unit (d = 90 mm, 0.22 μm, Merck, Millipore, Darmstadt, Germany). Precipitation of supernatant samples was carried out on ice for a period of 30 min, by adding 100% (*w*/*v*) TCA to 50 mL filtered final solution in the ratio 1:10. Precipitated material was recovered via centrifugation at 15,700 rcf for 15 min. The extracellular proteins in the resulting pellet were washed three times with ice-cold acetone (Merck, Millipore, Darmstadt, Germany), dried and dissolved in 30 µL of 25-mmol L^−1^ ammonium bicarbonate for further analysis. The total protein concentration was measured in precipitated samples using a BCA protein assay kit (Novagen, Madison, WI, USA) according to the instruction manual.

### 4.4. Assessment of Specific Proteolytic Activity

Specific proteolytic activity was determined with following substrates: Z–Ala–Ala–Leu–pNa for subtilisin-like proteinase and Z–Glu–pNa for glutamyl–endopeptidase as described in [40,41].

### 4.5. SDS-PAGE

Polyacrylamide gel electrophoresis (PAGE) was carried out using the MiniPROTEAN II electrophoretic cell device (BioRad, Hercules, CA, USA). Each protein sample (100 µg) was mixed with 50-mmol L^−1^ Tris-HCl buffer (pH 6.8) containing 2% SDS, 10% glycerol, 0.1% bromophenol blue and 100-mmol L^−1^ dithiothreitol (PAGE loading buffer) and heated for 5 min at 95 °C. After heat denaturation, samples were subjected to electrophoresis on 12.5% polyacrylamide gel. Gel was run under following conditions: 120 V/20 mA for approximately 2.5 h in 1 × SDS running buffer (20-mmol L^−1^ Tris base, 240-mmol L^−1^ glycine, pH 8.3). PageRuler unstained low range protein ladder (#26632 Thermo Scientific, Waltham MA, USA) was used as a protein reference. The total precipitated proteins were loaded into the a 10 mm gel for electrophoresis Gels were fixed in the fixation solution (consisting of isopropanol:acetic acid:water at 2:1:7 *v*/*v*/*v*) for 1 h and stained overnight with Coomassie G-250 Stain (Bio-Rad, Hercules, CA, USA). On the following day, gels were washed for 1 h in 10% acetic acid. Gel scanning was done by ImageQuant LAS 4000 (GE, Freiburg, Germany).

Degrees of proteases purity (757.11 for subtilisin-like proteinase and 1257 for glutamyl endopeptidase) used for the calibration curve construction were calculated according to the method described in [42,43]. Degree of purity expressed in arbitrary units.

### 4.6. In-Gel Tryptic Digestion and Peptide Extraction

Protein bands corresponding to the molecular mass of serine proteases (AprBp—27 kDa, GseBp—23 kDa) were excised from the gel, diced into 1 × 1 mm pieces and placed into a protein low-binding Eppendorf tubes (LoBind, Hamburg, Germany). Gel pieces were washed in 200 µL of ultrapure water (Merck, Millipore, Darmstadt, Germany). Coomassie dye was removed by 2–3 wash cycles with 200 µL mixture of 100% acetonitrile and 100-mmol L^−1^ ammonium bicarbonate at 1:1 *v*/*v*. Subsequently, gel pieces were incubated at room temperature in 100% acetonitrile until achieving a white appearance. Trypsin was then added at a ratio of 1:100 (enzyme:protein), *w*/*w* (stock concentration: 1 µg mL^−1^, dissolved in 25-mmol L^−1^ ammonium bicarbonate and 10% acetonitrile) and incubating overnight at 37 °C. Tryptic peptides were extracted from the gel pieces by 0.1% TFA solution. Next, samples were sonicated in an ultrasound bath for 5 min. The supernatant (containing tryptic peptides) was transferred to a clean 0.5-mL tube (BioRad, Hercules, CA, USA). The gel pieces were again extracted with an additional 50 µL of 0.1% TFA solution and sonication for 5 min, and the supernatant was added to the previous extractions in the 0.5-mL tube. The pooled extracted peptides were dried by SpeedVac (Eppendorf, Hamburg, Germany). A mobile phase A (water:acetonitrile:formic acid 95:5:0.1 *v*/*v*/*v*) was introduced into each tube in amounts to achieve the desired peptide concentrations and gently agitated by vortexing. Peptides solution was transferred to a vial for liquid chromatography-mass spectrometric multiple reaction monitoring (LC-MRM-MS) analysis.

### 4.7. In Silico Peptide Selection and Skyline Settings

For quantitative evaluation of *B. pumilus* serine proteases during MRM assay, unique peptides for AprBp and GseBp were selected using the Skyline software (version 20.1, 64-bit, Seattle, WA, USA) in accordance with the setting described by Kumar et al. (2016) [29]. *In silico* digestion was achieved by using the following parameters in "peptide settings" (settings > peptide settings): enzyme: trypsin; peptide length: 8–25 amino acids (aa) long (peptide sequences less than 7 aa are not very selective or unique for the desired protein and peptides with more than 25 aa would be more difficult to extract from gel); missed cleavages: none. For fragment selection, peptides that contained cysteine and methionine were excluded from the analysis due to their possible modification. For MRM analysis the following parameters were used "transition settings" (settings > transition settings): precursor charges: 2; fragment ion charges: 1; ion type: y, b; product ions: 3.

### 4.8. Quantitative LC–MRM–MS Analysis

All trypsin digested samples were analyzed by high-performance liquid chromatography (Infinity1290, Agilent, Santa Clara, CA, USA)– electrospray ionization mass spectrometry in MRM mode using 6500 Qtrap (Sciex, Toronto, ON, Canada) at unit resolution with a dwell time of 20 ms. Separations were performed on Titan C_18_ column, 100-mm-long × 2.1 mm i.d., packed with 1.9 μm particles (Supelco, Milford, MA, USA) using a flow rate of 0.4 mL min^−1^ with a 15-min total run and gradient of B phase from 0% to 30%. The mobile phases comprised (A) water containing 5% acetonitrile with 0.1% formic acid and (B) acetonitrile containing 5% water with 0.1% formic acid. The source parameters used in the study were capillary voltage 5.2 kV, drying gas−1 pressure 60 psi, drying gas−2 pressure 60 psi, curtain gas pressure 35 psi, temperature 500 °C. Collision energy and declustering potential was calculated for each peptide using the Skyline software ver. 20.1 (version 20.1, 64-bit, Seattle, WA, USA). 

## Figures and Tables

**Table 1:** Precursor ions and their transitions were used for final multiple reaction monitoring (MRM) assay.

Precursor Ion Q1, *m*/*z*	Ion Charge	Product Ion Q3, *m*/*z*	Peptide	Declustering Potential (V)	Collision Energy (V)	Retention Time (min)
**AprBp**						
521.27		802.42	APAVHAQGYK	69.1	27.6	1.03
	+2	703.35				
		566.29				
487.73		860.42 *	NAVDTANNR	66.7	26.4	0.79
	+2	690.31				
		575.28				
887.93		1145.56	LENTATPLGNSFYYGK	95.8	40.8	5.62
	+2	1048.50				
		935.42				
**GseBp**						
655.82		993.51 *	TDTNIGNTVGYR	78.9	32.5	1.39
	+2	766.38				
		709.36				
418.71		723.33	ISGYPGDK	61.6	23.9	1.11
	+2	636.29				
		416.21				
722.87		1130.58	ATAAFVEFINYAK	83.8	34.9	6.46
	+2	983.51				
		884.45				

* Peptides used for quantification.

**Table 2:** Quantification of serine proteases AprBp and GseBp in the optimized LIKE expression system by MRM analysis. SN—supernatant; bacitracin is the inducer (50 µg mL^−1^); "−"—without addition of the inducer; "+"—after addition of the inducer. Strains *B. pumilus* 3–19 and *B. subtilis* AT1 were grown without the inducer (bacitracin).

Strain	Total Protein Concentration (μg mL^−1^) in SN, Inducer "−"	Total Protein Concentration (μg mL^−1^) in SN, Inducer "+"	Target Protein Concentration (μg mL^−1^) in the Vial, Inducer "−"/"+"	Target Protein Concentration (μg mL^−1^) in SN, Inducer "−"/"+"
**Subtilisin-like protease (AprBp)**				
*B. pumilus* 3–19	4.5	–	6	1.63
*B. subtilis* AT1	0.32	0.33	0	0
*B. subtilis* MRB044 (SP*_AprBp_*)	2	5	0.2/5	0.024/1.5
*B. subtilis* MRB046 (SP_Yngk_)	1.3	1.8	0.45/0.25	0.005/0.03
**Glutamyl endopeptidase (GseBp)**				
*B. pumilus* 3–19	4.5	–	5	1.3
*B. subtilis* AT1	0.32	0.33	0/0	0/0
*B. subtilis* MRB047 (SP*_GseBp_*)	1.2	1.65	0.005/0.05	0.00036/0.005
*B. subtilis* MRB049 (SP_Yngk_)	2.06	3.2	0.05/0.3	0.006/0.06

Production of both serine proteases in native *B. pumilus* 3–19 strain was higher than in all constructed recombinant *B. subtilis* strains.

**Table 3:** Bacterial strains used in this study.

Strain	Relevant Genotype	Source
*B. pumilus* 3–19	Str^R^	Laboratory of Biosynthesis and Bioengineering of Enzymes, KFU
*B. subtilis*:		
BG2036	Δ*aprE-*684*,* Δ*nprE*522	(Yang et al [39])
AT1	Δ*aprE-*684*,* Δ*nprE*522*,* pLIKE-rep	(Tikhonova et al [23])
MRB044	pLIKE-rep + SP*_AprBp_* + *AprBp*	(Tikhonova et al [23])
MRB046	pLIKE-rep + SP_Yngk_ + *AprBp*	(Tikhonova et al [23])
MRB047	pLIKE-rep + SP*_GseBp_* + *GseBp*	(Tikhonova et al [23])
MRB049	pLIKE-rep + SP_Yngk_ + *GseBp*	(Tikhonova et al [23])

R-resistant.

## Supplementary Materials

Supplementary Materials can be found at https://www.mdpi.com/1422-0067/21/14/4924/s1.

## Abbreviations

AprBp	Subtilisin-like protease
DTT	Dithiothreitol
ELISA	Enzyme-linked immunosorbent assay
GseBp	Glutamyl endopeptidase
GRAS	Generally recognized as safe
IAA	Iodoacetamide
IPTG	Isopropyl β-galactopyranoside
iTRAQ	Isobaric tags for relative and absolute quantitation
LB	Lysogeny broth
LC–MS	Liquid chromatography-tandem mass spectrometry
LIKE	LIa-kontrollierte expression
LPS	Lipopolysaccharide
MRM	Multiple reaction monitoring
OD	Optical density
QQQ	Triple-quadrupole
Q1	First quadrupole
Q2	Second quadrupole
Q3	Third quadrupole
rcf	Relative centrifugal field
rpm	Revolutions per minute
SILAC	Stable isotope labeling by amino acids in cell culture
SN	Supernatant
TCA	Trichloroacetic acid
