# Supplementary material for: Label-Free Multiple Reaction Monitoring, a Promising Method for Quantification Analyses of Specific Proteins in Bacteria
Source: Int J Mol Sci. 2020 Jul 12;21(14):4924. doi: 10.3390/ijms21144924 (PMC7404251; doi:10.3390/ijms21144924)
Supplement: Supplementary file 1 [file ijms-21-04924-s001.pdf]

Supplementary materials

Table S1. MRM transitions used for initial screening of serine proteases (AprBp and GseBp) of *B. pumilus* are listed. The analysis was carried out by Skyline and the declustering potential and collision energy are shown. At least three different peptides with three fragment ions were considered for final analysis (gray highlighted). The most effective transition was used for quantification.

Precursor ion Q1, m/z	Product ion Q3, m/z	Peptide	Declustering Potential (V)	Collision Energy (V)
		**AprBp**		
261.13	434.22	SASEK.+2y4		
261.13	363.18	SASEK.+2y3	50.2	18.3
261.13	276.15	SASEK.+2y2		
407.2	726.41	SYIVGFK.+2y6		
407.22	563.35	SYIVGFK.+2y5	60.8	23.5
407.22	450.27	SYIVGFK.+2y4		
451.73	774.41	QAVTQNGGK.+2y8		
451.73	703.37	QAVTQNGGK.+2y7	64	25.1
451.73	604.30	QAVTQNGGK.+2y6		
451.73	503.25	QAVTQNGGK.+2y5		
428.76	743.44	LINAAQVK.+2y7		
428.76	630.35	LINAAQVK.+2y6	62.4	24.3
428.76	516.31	LINAAQVK.+2y5		
428.76	445.27	LINAAQVK.+2y4		
891.42	1190.56	LEHDPSIAYVEEDHK.+2y10		
891.42	1103.53	LEHDPSIAYVEEDHK.+2y9	96.1	40.9
891.42	990.45	LEHDPSIAYVEEDHK.+2y8		
891.42	919.41	LEHDPSIAYVEEDHK.+2y7		
874.96	1115.64	AEAYAQTVPYGIPQIK.+2y10		
874.96	1014.59	AEAYAQTVPYGIPQIK.+2y9	94.9	40.4
874.96	915.52	AEAYAQTVPYGIPQIK.+2y8		
874.96	485.30	AEAYAQTVPYGIPQIK.+2y4		
521.27	970.51	APAVHAQGYK.+2y9		
521.27	873.45	APAVHAQGYK.+2y8		
521.27	802.42	APAVHAQGYK.+2y7	69.1	27.6
521.27	703.35	APAVHAQGYK.+2y6		
521.27	566.29	APAVHAQGYK.+2y5		
244.64	431.26	GANVK.+2y4		
244.64	360.22	GANVK.+2y3	49	17.7
244.64	246.18	GANVK.+2y2		
487.73	860.42*	NAVDTANNR.+2y8		
487.73	789.38	NAVDTANNR.+2y7	66.7	26.4
487.73	690.31	NAVDTANNR.+2y6		
487.73	575.28	NAVDTANNR.+2y5		
1119.07	1168.58	GVVVVAAAGNSGSTGSTSTVGYPAK.+2y12	112.7	49.1
1119.07	315.20	GVVVVAAAGNSGSTGSTSTVGYPAK.+2y3		
887.93	1145.56	LENTATPLGNSFYYGK.+2y10		
887.93	1048.50	LENTATPLGNSFYYGK.+2y9	95.8	40.8
887.93	935.42	LENTATPLGNSFYYGK.+2y8		
479.75	788.38	GLINAQAASN.+2y8		
479.75	675.30	GLINAQAASN.+2y7	66.1	26.1
479.75	561.26	GLINAQAASN.+2y6		
479.75	490.22	GLINAQAASN.+2y5		
		**GseBp**		
655.82	993.51*	TDTNIGNTVGYR.+2y9		
655.82	879.46	TDTNIGNTVGYR.+2y8	78.9	32.5
655.82	766.38	TDTNIGNTVGYR.+2y7		
655.82	709.36	TDTNIGNTVGYR.+2y6		
418.71	723.33	ISGYPGDK.+2y7		
418.71	636.29	ISGYPGDK.+2y6	61.6	23.9
418.71	579.27	ISGYPGDK.+2y5		
418.71	416.21	ISGYPGDK.+2y4		
722.87	1130.58	ATAAFVEFINYAK.+2y9		
722.87	983.51	ATAAFVEFINYAK.+2y8	83.8	34.9
722.87	884.45	ATAAFVEFINYAK.+2y7		
722.87	755.40	ATAAFVEFINYAK.+2y6		

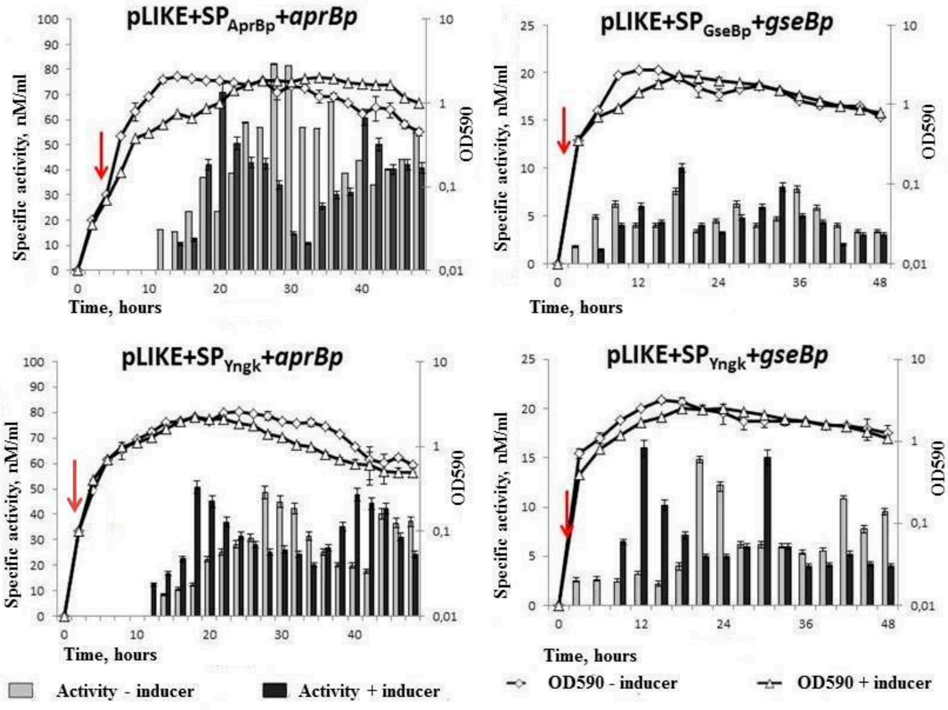

Figure S1. Growth and specific activity of recombinant strains. Growth profiles are shown by symbols: (◊) without inducer, (Δ) with bacitracin. Vertical arrow indicates time point of bacitracin addition (final conc. 50 µg/ml; OD600~0.4-0.5). The samples were at incubation of 48 h in 500 mL shake-flask, every 2 h 1 ml from shake-flask were collected and centrifuged at 13,000×g for 1 min. After centrifugation, the supernatant was used for analysis of specific proteolytic activity. The specific activity was converted to 1 nM of p-nitroanalide (pNA) per min (U) from specific substrate (Z-Glu-pNa for glutamyl endopeptidase; Z-Ala-Ala-Leu-pNa for subtilisin like proteinase). Each diagram represents the mean of two parallel experiments, growth and activity with inducer (bacitracin final conc. 50 µg/mL) and without inducer. Error bars represent standard error.

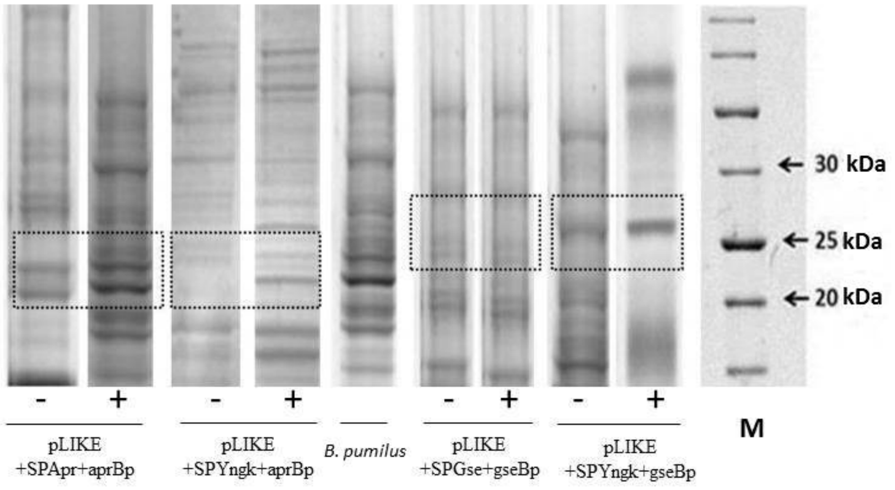

Figure S2. SDS-PAGE of culture SN from recombinant strain *B. subtilis* and *B. pumilus,* incubated in LB medium. Lane M, broad-range size marker (#26614, Thermo Scientific, USA).
